# Fabry cardiomyopathy presenting as hypertrophic phenotype with left ventricular outflow tract obstruction: a case series

**DOI:** 10.3389/fcvm.2026.1901732

**Published:** 2026-08-11

**Authors:** Felycia Fernanda Hosyanto, Yuanzhu Li, Suxin Luo, Bi Huang

**Affiliations:** Department of Cardiology, The First Affiliated Hospital of Chongqing Medical University, Chongqing, China

**Keywords:** case series, differential diagnosis, Fabry cardiomyopathy, left ventricular hypertrophy (LVH), left ventricular outflow tract obstruction

## Abstract

**Background:**

Fabry disease is a cardiomyopathy with multisystemic manifestations that is easily to be misdiagnosed as another type of cardiomyopathy. Fabry cardiomyopathy may present with left ventricular outflow tract obstruction (LVOTO), mimicking obstructive hypertrophic cardiomyopathy (oHCM). We present three cases of genetically confirmed Fabry disease that were initially misdiagnosed as oHCM, highlighting the importance of early recognition and tailored management.

**Methods:**

We performed Fabry disease screening on patients diagnosed with oHCM and ultimately identified three individuals who have received oHCM-specific treatments including mavacamten and alcohol septal ablation.

**Results:**

Three patients (2 females, 1 male; aged 48–66 years) who were initially diagnosed with and managed for oHCM based on septal hypertrophy and elevated left ventricular outflow tract gradients (LVOTG), was found to have GLA gene mutations. Enzyme replacement therapy with agalsidase alfa in two patients was associated with stabilization or improvement in wall thickness and LVOTO.

**Conclusion:**

Fabry cardiomyopathy can present with a hypertrophic phenotype and significant LVOTO, and is often misdiagnosed as oHCM. This further underscores the importance of genetic testing in the etiological diagnosis of hypertrophic cardiomyopathies. Enzyme replacement therapy may ameliorate obstruction, while mavacamten and septal reduction therapies require caution without definitive genotype-phenotype correlation.

## Introduction

Fabry disease is one rare X-linked recessive lysosomal storage disorder due to GLA gene mutation that leads to the accumulation of globotriaosylceramide (Gb3) in numerous tissues, with the heart as the most commonly affected organ (1). Left ventricular hypertrophy (LVH) is the most common and main characteristic of cardiac involvement in Fabry disease (2), that is typically concentric and non-obstructive (2). However, obstructive type may present in minority cases. When left ventricular outflow tract obstruction (LVOTO) is present, the clinical, imaging, and hemodynamic features overlap significantly with those of obstructive hypertrophic cardiomyopathy (oHCM), which is the primary reason for misdiagnosis. The treatment strategies for the two conditions differ fundamentally: specific therapies for oHCM have limited efficacy in Fabry cardiomyopathy, and delayed diagnosis may lead to disease progression. Therefore, improving the recognition of the hypertrophic–obstructive phenotype of this disease is crucial. Here we report a case series of patients initially presenting with oHCM, who were later diagnosed with Fabry disease through genetic testing.

## Case series

### Case 1

**Figure 1:**
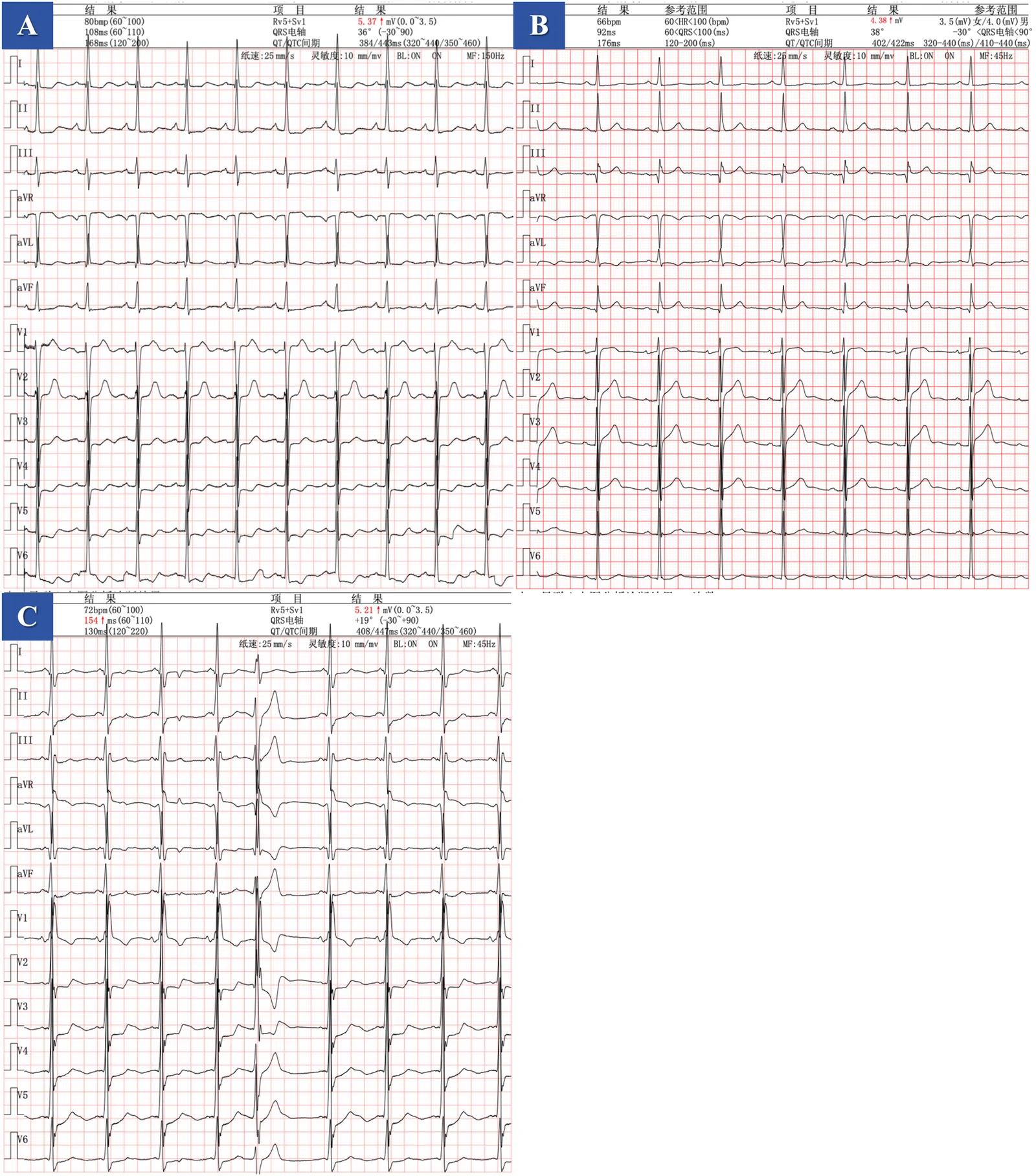
ECG showing high electrical voltage for patient 1 **(A)**, patient 2 **(B)**, and patient 3 **(C)**.

A 54-year-old female presented with chest tightness, accompanied by chest pain that radiated to the back. The patient denied symptoms such as fingertip pain, hypohidrosis, or cutaneous gliomas. Initial echocardiography showed asymmetric septal hypertrophy with LVOTO [resting left ventricular outflow tract gradients (LVOTG) of 79 mmHg]. Patient was diagnosed with oHCM and was prescribed angiotensin receptor-neprilysin inhibitor (ARNI) and calcium channel blocker (CCB), yet symptoms did not improve significantly. Therefore, patient underwent a repeat echocardiogram and electrocardiogram (ECG), showing resting obstructive septal hypertrophy and left ventricular high voltage along with ST-T changes, respectively (Figure 1). Besides, N-terminal pro-brain natriuretic peptide (NT-proBNP) was 961 pg/mL (reference range of 0–220 pg/mL) and cardiac troponin level was within normal range. This patient was prescribed mavacamten 2.5 mg once daily and underwent GLA gene testing, which revealed Fabry disease, with the mutation at chrX:100654460 (c.640-526C > T), and 2.55 μmol/L/h of GLA enzyme (reference range of 2.20–17.65 μmol/mL). Although the patient received a definitive diagnosis, she refused to undergo enzyme replacement therapy (ERT) treatment and did not follow up regularly. Her daughter also underwent GLA gene testing revealing the same mutation location.

### Case 2

**Figure 2:**
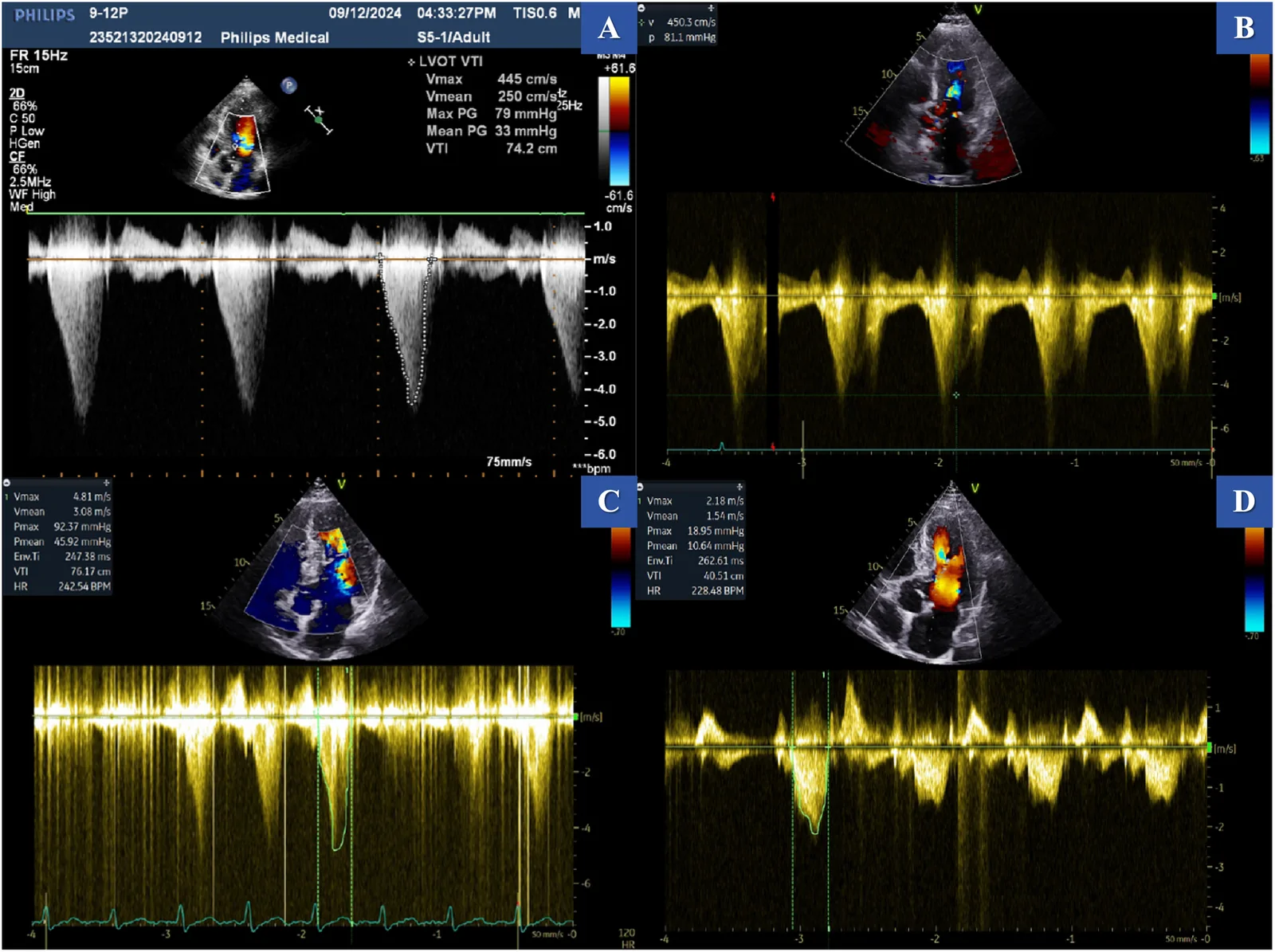
Echocardiography findings of asymmetrical septal hypertrophy with left ventricular outflow tract stenosis or obstruction **(A,B)**, and stress echocardiography showing latent obstruction **(C,D)**.

A 48-year-old male was admitted due to recurrent dizziness and exertional dyspnea, accompanied with syncope for 3 times. Meanwhile, he complained of intermittent fingertip pain. Cardiac ischemic biomarkers, NT-proBNP, and renal function results were in reference range. ECG showed elevated QRS electrical voltage and T wave abnormality (Figure 1), echocardiography showed left ventricular hypertrophy (LVH) with LVOTO, resting LVOTG of 19mmHg, and exercise LVOTG of 92 mmHg (Figure 2). He underwent coronary angiography (CAG) showing complete occlusion of the circumflex artery and severe stenosis of the left anterior descending artery. Therefore, this patient received antiplatelets, beta-blocker, ARNI, and lipid lowering medications. This patient underwent GLA gene testing and mutation was found at chrX:100655692 (c.601T > G) with GLA enzyme of 2.18 μmol/L/h. Agalsidase alfa was initiated after Fabry disease was genetically confirmed and the patient's LVOTG showed significant reduction after ERT for 10 months. The ECG, echocardiography, NT-proBNP, and cardiac ischemic biomarkers did not show significant changes during the follow-up. His daughter also underwent GLA gene testing and was found to be genetically positive.

### Case 3

A 66-year-old female complained of exertional dyspnea for more than 1 year that worsened for 2 months, along with exertional chest tightness. The patient reported no fingertip pain, hypohidrosis, or other symptoms. At admission, the BNP was 143 pg/mL and cardiac ischemic biomarkers were within normal range. ECG indicated LVH, ST-T changes, and supraventricular premature beat (Figure 1). Patient underwent CAG and showed mild stenosis in left anterior descending artery, therefore, she received secondary prevention of coronary heart disease. Despite this, the symptoms still deteriorated. Cardiac troponin was markedly elevated, approximately 270 times the upper limit of normal and NT-proBNP was 3,770 pg/mL. ECG suggested LVH and right bundle branch block, while echocardiography demonstrated septal hypertrophy with LVOTO 73mmHg at rest. This patient was diagnosed with oHCM and received alcohol septal ablation and the LVOTO reduced to 26 mmHg after alcohol septal ablation. However, two years later, LVOTO increased to 81 mmHg and she was administered with mavacamten 2.5 mg once daily, however, she still exhibited exertional dyspnea. Finally, the patient underwent GLA gene testing, which revealed a positive result for Fabry disease. Mutation was found at chrX:100661470_100661472 (c.194 + 1226_194 + 1228del) and GLA enzyme was 3.37 μmol/L/h. Agalsidase alfa was initiated and echocardiography follow-up was conducted, showing improvement of echocardiographic findings. There were no significant changes in ECG, echocardiography, and NT-proBNP during the follow-up. The same mutation location was found in the patient's daughter.

**Figure 3:**
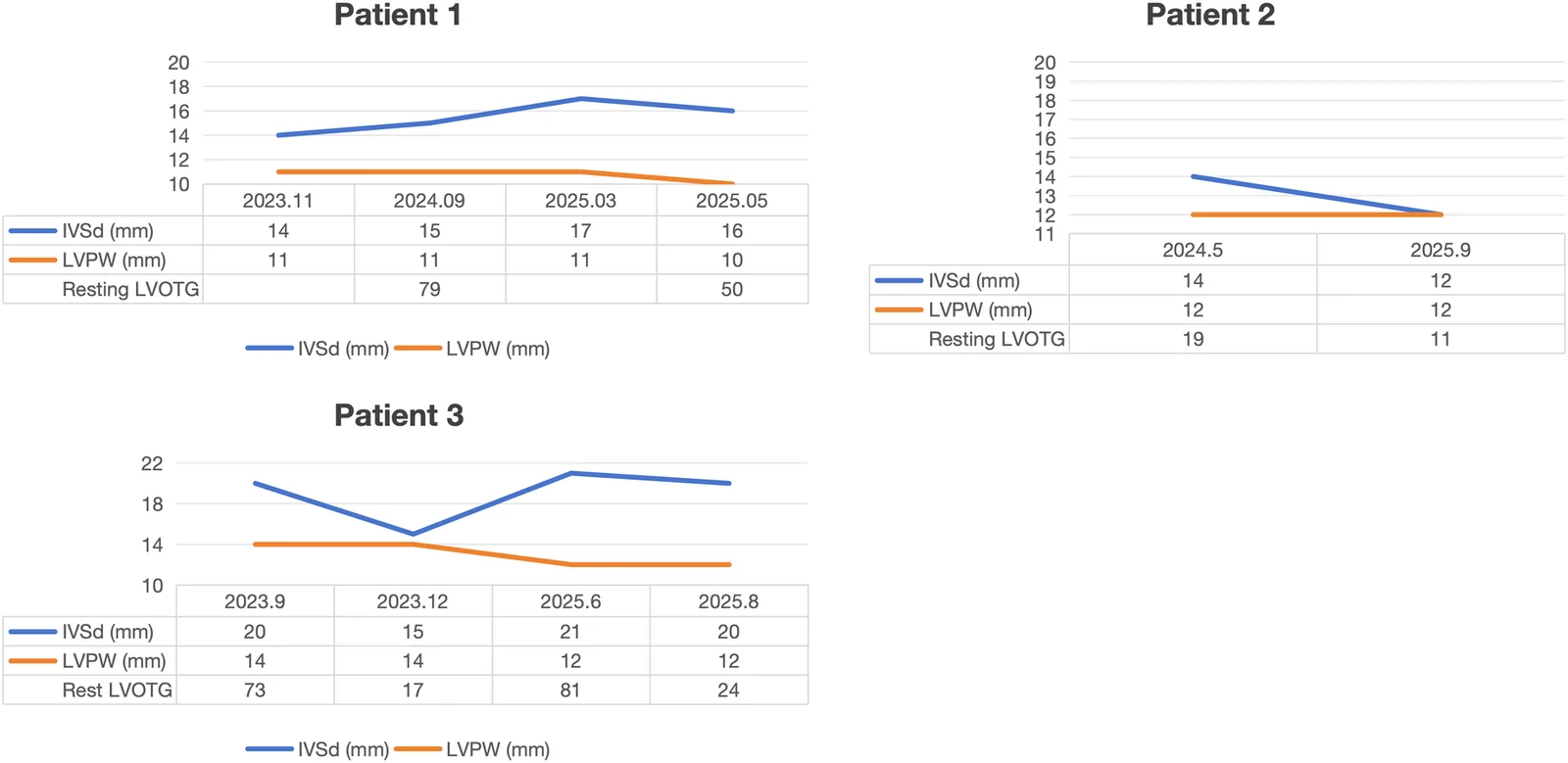
Changes of interventricular septal end-diastolic thickness (IVSd), left ventricular posterior wall end-diastolic thickness (LVPWd), and resting left ventricular outflow tract gradient (LVOTG) in all three patients.

**Figure 4:**
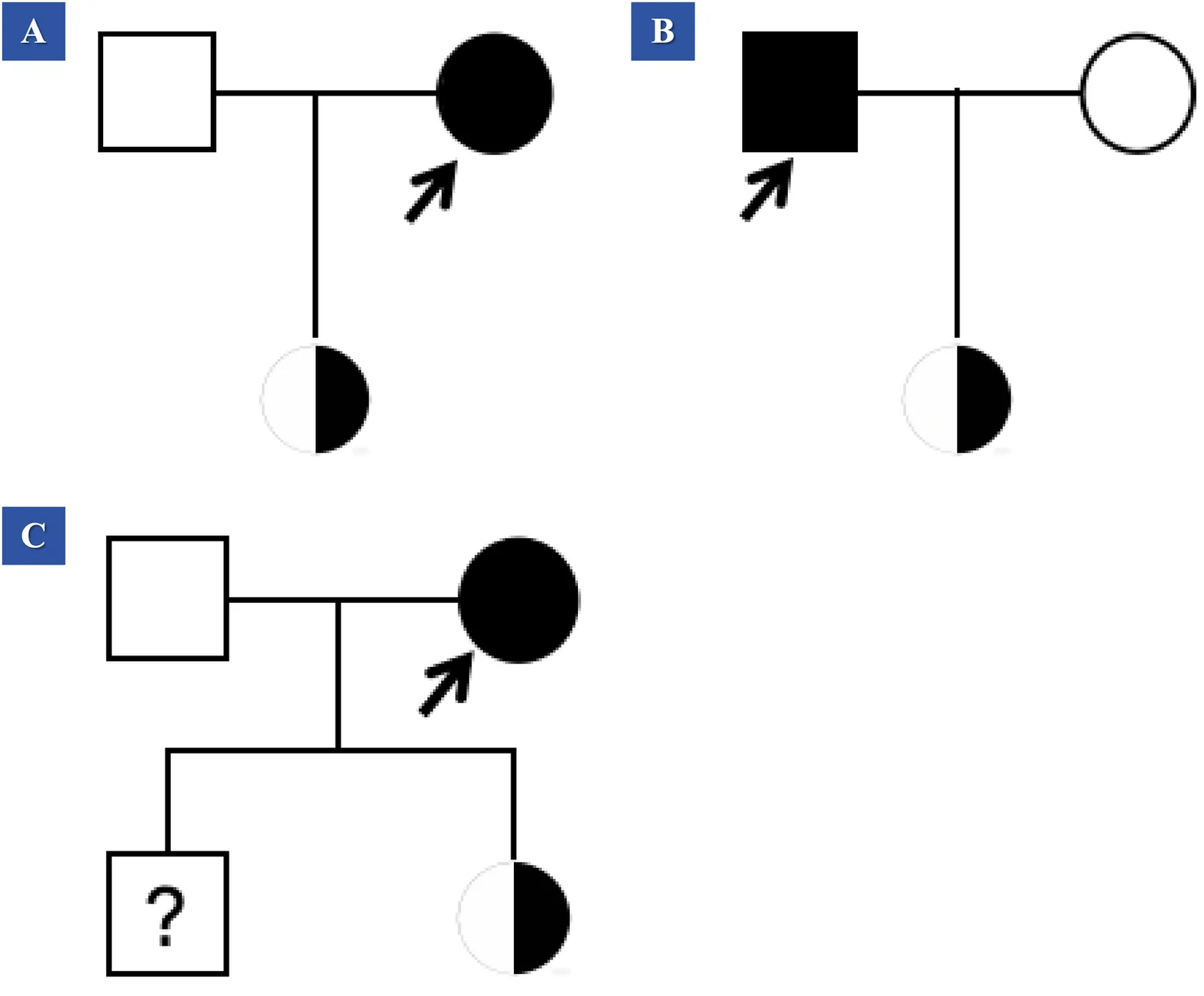
Family lineages of patient 1 **(A)**, patient 2 **(B)**, and patient 3 **(C)**.

The changes in the echocardiograms of the three patients are shown in Figure 3 and their family lineages are shown in Figure 4.

## Discussion

We present three cases of genetically confirmed Fabry disease initially misdiagnosed as oHCM, all of whom exhibited significant LVOTO and received oHCM-specific therapies before the correct diagnosis was established. This case series highlights several critical clinical lessons. First, Fabry cardiomyopathy can present with a true obstructive phenotype. Fabry disease leads to progressive accumulation of Gb3 in cardiomyocytes, conduction system cells, and vascular endothelial cells (3). Although Fabry cardiomyopathy is typically concentric and non-obstructive (2), as demonstrated in our series, a minority of patients develop asymmetric septal hypertrophy with LVOTO, creating a phenotypic mimic of oHCM that is easily misdiagnosed. In our cases, the presence of septal hypertrophy and resting LVOTG ranging from 59 to 92 mmHg fulfilled conventional echocardiographic criteria for oHCM, leading to initial management with beta-blockers, CCB, ARNI, and even mavacamten or alcohol septal ablation.

The association between Fabry disease and LVOTO is not without precedent. Several studies have previously documented obstructive phenotypes in Fabry cardiomyopathy (4–7). Our case series confirms and extends these prior observations by documenting LVOTG in three patients, all of whom were initially misdiagnosed as oHCM. Our series uniquely highlights the sex-based differences in atypical presentation and the dangers of phenotype-directed therapies in the absence of precise cause confirmation.

The mechanism of LVOTO in Fabry cardiomyopathy remains incompletely understood (2). Unlike sarcomeric HCM, where obstruction arises from septal hypertrophy, systolic anterior motion of the mitral valve, and elongated mitral leaflets (8), Fabry-related LVOTO may result from a combination of asymmetric septal thickening and altered left ventricular geometry (9). Histopathologically, Gb3 deposition may preferentially involve the basal septum in some patients, creating a focal obstructive phenotype (2, 9). Notably, two of our three patients also had concomitant coronary artery disease, complicating the clinical manifestation of Fabry disease (2), contributing to regional wall motion abnormalities and further complicated the hemodynamic picture.

A key finding from our series is the diagnostic delay associated with the obstructive phenotype. All three patients were initially managed as oHCM for variable periods, and oHCM-specific treatments were instituted before genetic testing was pursued. This delay is clinically consequential because the natural history of Fabry cardiomyopathy differs from that of oHCM. Fabry disease progresses inexorably toward heart failure, arrhythmias, and sudden cardiac death (2). Importantly, the therapies that are effective in reducing LVOTG in oHCM do not address the underlying pathophysiology of Gb3 accumulation. In Case 1, mavacamten was prescribed without clear benefit, and in Case 3, alcohol septal ablation was performed followed by mavacamten, yet symptoms persisted. These observations underscore the principle that phenotype-directed therapy without precise cause confirmation can be ineffective or potentially harmful.

Genetic testing is indispensable in the etiological evaluation of HCM. The role of GLA gene testing cannot be overstated. Current guidelines recommend genetic testing for all patients with unexplained LVH (8–10), particularly when atypical features are present, such as angiokeratomas, acroparesthesias, hypohidrosis, cornea verticillata, and neuropathies (2, 3, 9). However, as our cases demonstrated, Fabry disease may present with isolated cardiac involvement, lacking classic multisystemic manifestations, especially in female patients (Cases 1 and 3). This is categorized as atypical Fabry disease, which is observed more frequently in females than in males (2). In contrast, Patient 2 exhibited both cardiovascular and nervous system involvement, consistent with the classic multisystemic involvement. Therefore, we advocate for broader utilization of GLA gene testing in all adult patients presenting with LVH, regardless of the presence or absence of LVOTO, particularly when wall thickness is disproportionate to hypertension or when electrocardiography shows high voltage with relatively mild hypertrophy, a subtle clue often overlooked.

Among the three variants identified in our patients, c.601T > G, known as p.Phe201Val for its protein-level name (11), is a well-known pathogenic mutation in the GLA gene which is closely associated with Fabry disease, including cardiovascular involvement [e.g., LVH (12), arrhythmias (13)] and multisystem involvement [e.g., renal, neurologic, and dermatologic (14)]. There are no prior literatures regarding the other two variants, c.640-526C > T and c.194 + 1226_194 + 1228del. Although these two variants are located in intronic regions, they may potentially lead to pathogenicity by disrupting normal mRNA splicing. Furthermore, both patients exhibit corresponding clinical phenotypes, suggesting that these variants may be deleterious. However, further genetic and functional evidence is required to confirm their clinical significance.

The third key point of our case series is that ERT may ameliorate LVOTO, whereas conventional oHCM therapies carry risks without a definitive genotype-phenotype correlation. ERT with agalsidase alfa or agalsidase beta remains the cornerstone of disease-specific therapy for Fabry cardiomyopathy (15). In Cases 2 and 3, agalsidase alfa treatment was associated with stabilization or improvement in left ventricular wall thickness and reduction in LVOTG. This observation, while not proof of causality in a case series, aligns with prior reports suggesting that ERT may reduce myocardial Gb3 storage, improve diastolic function, and potentially modify ventricular geometry, thereby alleviating dynamic obstruction (16). Importantly, ERT is most effective when initiated before irreversible fibrosis develops, as detected by late gadolinium enhancement on cardiac magnetic resonance (17).

The use of mavacamten, a cardiac myosin inhibitor approved for oHCM, in Fabry cardiomyopathy requires caution. Mavacamten reduces LVOTG by decreasing hypercontractility (18), but it does not reduce Gb3 accumulation, which requires ERT (15). Theoretical concerns include the potential for mavacamten to unmask underlying systolic dysfunction in Fabry disease, which may coexist with LVH due to concomitant coronary microvascular disease or fibrosis. Mavacamten has been shown to cause a dose-dependent reduction in left ventricular ejection fraction in patients with obstructive HCM (18), and Fabry disease is frequently associated with myocardial fibrosis and coronary microvascular dysfunction that can impair systolic reserve (2). Until prospective data are available, our case series indicated mavacamten should not be routinely used in Fabry cardiomyopathy with obstructive phenotype, and early ERT is the optimal strategy once Fabry disease is diagnosed.

This case series has several limitations. First, the diagnosis of Fabry disease requires a comprehensive assessment, including clinical manifestations, echocardiography, ECG, and other imaging modalities, as well as *α*-galactosidase A activity, GLA gene mutation analysis, and elevated plasma Lyso-Gb3 levels. When necessary, tissue biopsy is required for pathological confirmation. Although these patients were genetically diagnosed with Fabry disease, there is a lack of tissue biopsy evidence, particularly endomyocardial biopsy. Second, some patients may harbor co-existing sarcomeric gene variants (19, 20); however, as our patients did not undergo whole-exome sequencing, we cannot rule out the coexistence of Fabry disease and sarcomeric gene variant-related disorders. Third, the duration of ERT was relatively short, and longer follow-up is required to evaluate the impact of ERT on cardiac structure, function, and electrophysiology in patients with Fabry disease.

## Conclusion

Fabry cardiomyopathy can present with an oHCM phenotype, leading to diagnostic delay. Genetic testing for GLA mutations should be considered in patients with unexplained LVH, regardless of the presence or absence of obstruction. ERT may ameliorate LVOTO and should be initiated promptly upon diagnosis, whereas oHCM-specific therapies including mavacamten and septal reduction require careful deliberation in the absence of definitive genotype-phenotype correlation. Early recognition of Fabry disease as a phenocopy of oHCM is essential to prevent inappropriate treatment and to enable timely disease-specific therapy.

## Data Availability

The raw data supporting the conclusions of this article will be made available by the authors, without undue reservation.
